# Animal Detection Precedes Access to Scene Category

**DOI:** 10.1371/journal.pone.0051471

**Published:** 2012-12-10

**Authors:** Sébastien M. Crouzet, Olivier R. Joubert, Simon J. Thorpe, Michèle Fabre-Thorpe

**Affiliations:** 1 Université de Toulouse, UPS, CerCo, Toulouse, France; 2 CNRS, UMR 5549, Toulouse, France; 3 Cognitive, Linguistic and Psychological Science, Brown University, Providence, Rhode Island, United States of America; National Institute of Mental Health, United States of America

## Abstract

The processes underlying object recognition are fundamental for the understanding of visual perception. Humans can recognize many objects rapidly even in complex scenes, a task that still presents major challenges for computer vision systems. A common experimental demonstration of this ability is the rapid animal detection protocol, where human participants earliest responses to report the presence/absence of animals in natural scenes are observed at 250–270 ms latencies. One of the hypotheses to account for such speed is that people would not actually recognize an animal *per se*, but rather base their decision on global scene statistics. These global statistics (also referred to as spatial envelope or gist) have been shown to be computationally easy to process and could thus be used as a proxy for coarse object recognition. Here, using a saccadic choice task, which allows us to investigate a previously inaccessible temporal window of visual processing, we showed that animal – but not vehicle – detection clearly precedes scene categorization. This asynchrony is in addition validated by a late contextual modulation of animal detection, starting simultaneously with the availability of scene category. Interestingly, the advantage for animal over scene categorization is in opposition to the results of simulations using standard computational models. Taken together, these results challenge the idea that rapid animal detection might be based on early access of global scene statistics, and rather suggests a process based on the extraction of specific local complex features that might be hardwired in the visual system.

## Introduction

There is clear evidence that humans are fast and efficient at detecting particular categories of objects such as animals and faces in natural scenes. This has been shown with a large range of protocols such as go/no-go categorization [1], [2], saccadic discrimination [3], [4], rapid serial visual presentations [5], [6] or change-detection tasks [7], using both behavioral and electrophysiological recordings [1], [8]. In rapid object categorization tasks the earliest responses (excluding anticipations) have been observed as early as 250 ms, suggesting purely feed-forward visual processing [1], [9], and demonstrating that a lot of information can be extracted from a new scene in the first couple of hundred milliseconds.

One way of interpreting these results would be to suppose that our brains have specific mechanisms for processing biologically important stimuli such as animals and faces [4], [7]. Alternatively, it could be that we are simply very good at using the whole image to extract meaning and guide object recognition [10]–[16]. Rapid access to scene category could rely on the extraction of 3D primitives [17], as well as global statistics about the spatial layout [11], [12], [14]. Because scene category can be extracted using very simple mechanisms, it might potentially be used to drive object recognition. There is already strong evidence that animal detection is easier when shown in an appropriate context [18]–[20]. For example, a cow is easier to detect when presented in a congruent scene such as a field, than when embedded in an incongruent scene, for example in a kitchen. When subjects are performing a classic go/no-go superordinate categorization task, these contextual effects can be seen from the very beginning of the reaction time distribution [18], that is to say, for behavioral responses occurring as early as 260 ms. Clearly, context can influence object recognition very early on, even with object categories that might be processed very fast such as animals. Studies using the go/no-go manual task have already shown that humans can categorize scenes (natural vs. man-made) of a flashed scene with a time-course similar to the one observed for object categorization [21]. But in such tasks a limit might have been reached as early manual responses are produced at similar latencies for animals, faces, vehicles and scene categories. In contrast, tasks using eye movements have demonstrated that saccades toward face targets can be triggered faster than with animal targets (100 ms vs. 120–130 ms) [4]. This raises the question of whether scene category can be processed fast enough to precede, and thus underlie or influence, animal detection.

This is the question that we attempted to answer in the present study using a saccadic choice task [3], [4] that allows to explore natural scene processing in a temporal window that is substantially earlier than using conventional behavioral tasks. In this saccadic choice task, two scenes are simultaneously presented on each side of a fixation cross and subjects are required to make a saccade as quickly and accurately as possible towards the target image.

**Figure 1:**
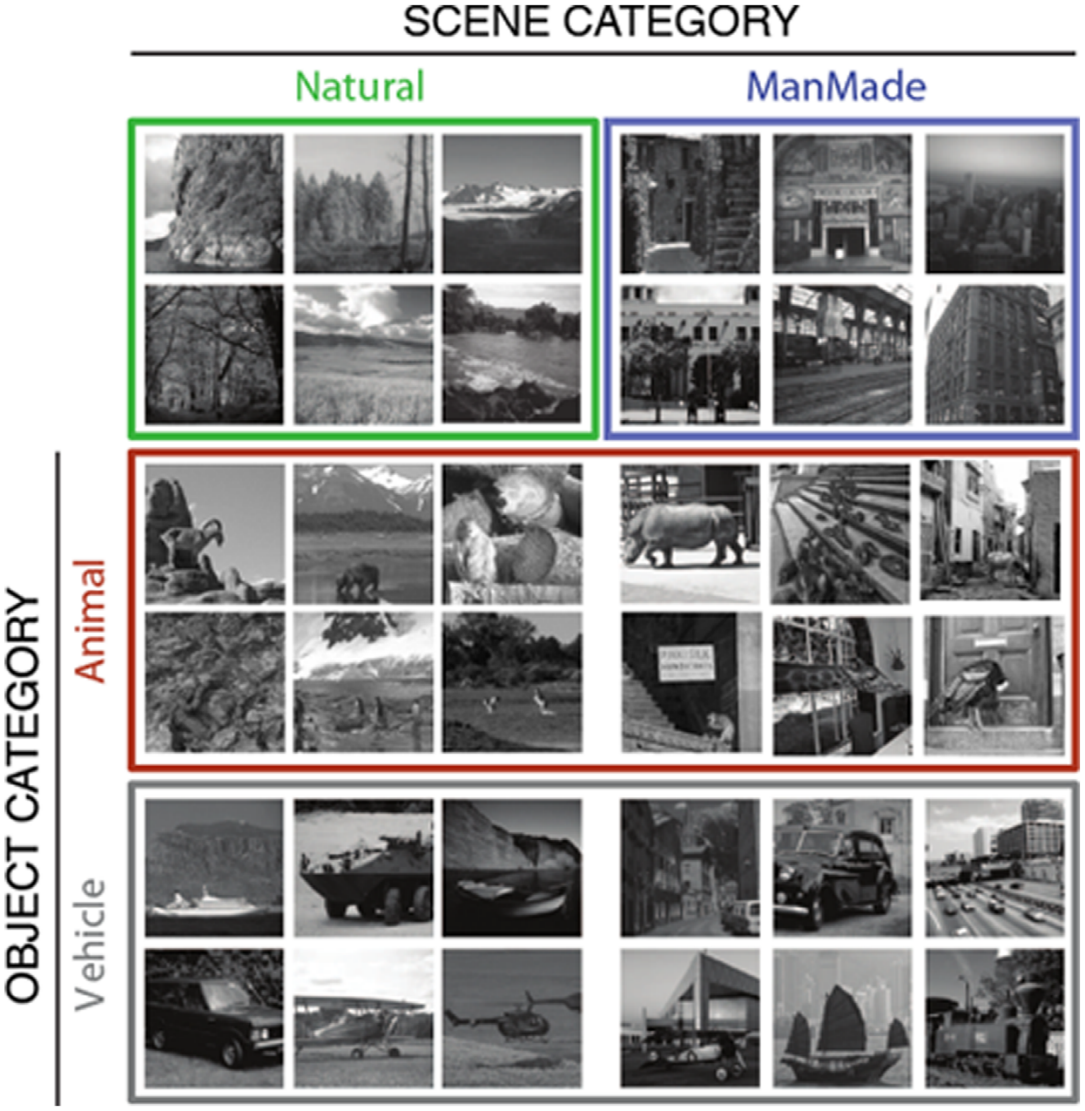
Examples of stimuli used in this study. In the two contextual discrimination tasks, only neutral natural and man-made environments (without foreground objects) were used as targets or distractors according to the task. In the two other object discrimination tasks, targets and distractors were animals or vehicles embedded (only original images were used, objects were never pasted) in either natural or man-made scenes. Average luminance and RMS contrast were equalized across the set of stimuli.

By using as targets two scene categories (“Man-made” and “natural”) and two object categories (“Animal” and “Vehicle”) in different blocks (Fig. 1), we evaluated the relative time needed to extract information about either different objects or different scene categories. The manipulation of the scene category (i.e. the background information) in the object task also allowed us to test interference effects from object/context congruency that had been documented in previous studies [18]–[20]. In this study, the photographs were not manipulated. Instead, contextual (in)congruence was obtained through the initial selection of images from a very large set. In addition, we also compared the behavioral results with the performance of standard computational models that have been suggested to account for the type of information that could be conveyed during the first feedforward sweep through the ventral visual stream.

The results showed that the earliest responses towards scene categories were produced at substantially longer latencies (160 ms) than with animal targets (120 ms) but were nevertheless earlier than saccades to the artefactual vehicle category (180 ms). In agreement with this first result, the first signs of context modulation in the animal detection task did not appear until 160 ms after image onset, i.e. the very same latency as the first reliable responses obtained for scene categories. Interestingly, simulations with a range of standard computational models predicted the opposite result, namely higher performance for scene than for object categorization.

## Results

### Comparing Object and Scene Discrimination

**Figure 2:**
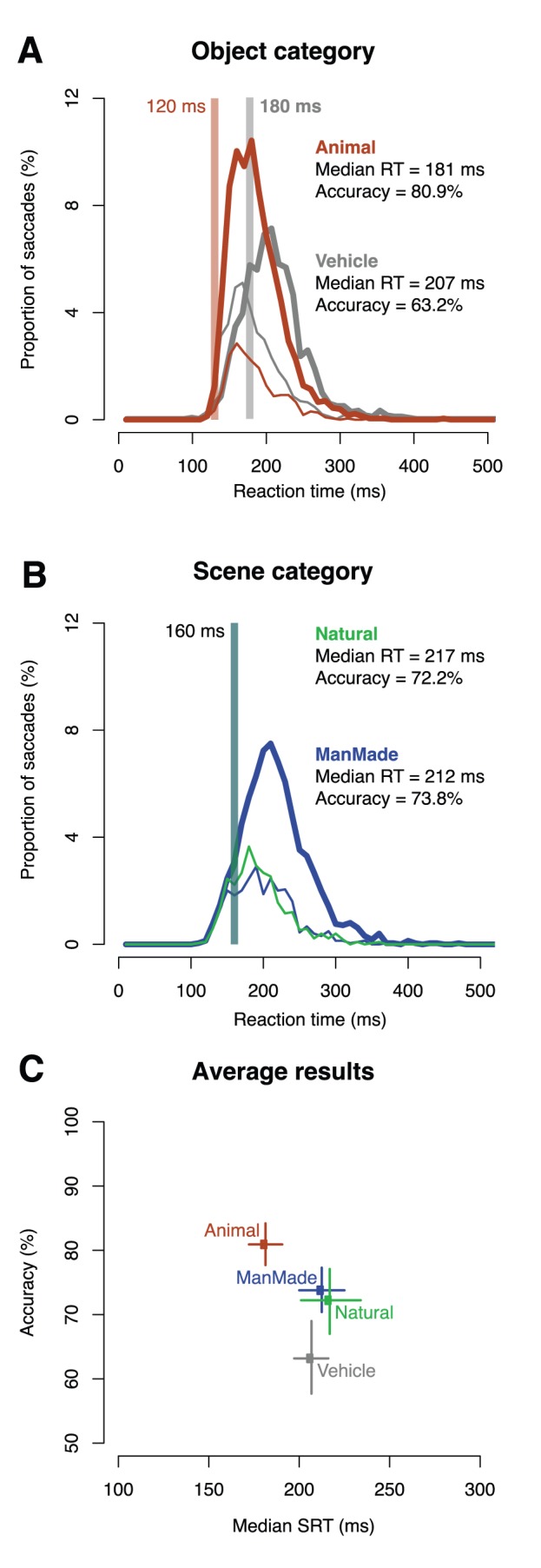
Behavioral results. (A, B) SRT distributions for correct (thick line) and incorrect (thin line) responses for each task with the percentage of responses pooled across all subjects and expressed over time using 10 ms time bins. Color code as in Figure 1 (animal target: brown, vehicle targets: Grey, Natural scene: green, man-made scene: Blue). A: SRT distributions confirm the object category asymmetry: animal responses (A, brown) are observed to be on average faster than vehicle ones (A grey) and with shorter minimal RT (respectively 120 and 180 ms) indicated by a grey thick vertical line. Note that saccades towards animals are difficult to control, since short latency saccades in the vehicle task are biased towards the scene that contained an animal (thin grey line). B: The time-course of scene processing is very similar in both scene tasks (man-made and natural), minimal RT indicated by a grey thick vertical line was found to be 160 ms in both cases. C: Accuracy as a function of median SRT for each task. Horizontal and vertical bars correspond to 95% confidence interval for median RTs and accuracies respectively. The values are computed using a percentile bootstrap with 2000 resamples.

Pairs of stimuli were displayed left and right of fixation and subjects had to make a saccadic response towards the target scene as fast and as accurately as possible. When the task was to target animals (regardless of the context of presentation), the participants were, on average, correct on 80.9% of the trials, with an averaged median saccadic reaction time (SRT) of 181 ms (Fig. 2C). Moreover, the minimal SRT (the first bin in which correct responses significantly outnumber errors using a χ2 test with a criterion of p<.05) computed on the group of subjects showed that fastest subjects were able to trigger saccades towards the correct side with SRTs as short as 120 ms (Fig. 2A). These results are comparable with those obtained by Kirchner & Thorpe [3] using the same animal category. Such high levels of performance were not observed using vehicles as targets. In that case, subjects scored only 63.2% with a median SRT of 207 ms and a minimal SRT of 180 ms (Fig. 2A–C, Table S1). Monte Carlo simulations (SI Methods) revealed that this advantage in favor of animals was highly significant (accuracy: p = 0.001, median RT: p<0.0001). Indeed, with vehicle targets, there was a clear tendency for short latency saccades to go in the wrong direction, i.e. towards the animal (Fig. 2A), whereas reliable saccades toward the vehicle target were only seen later on.

When performing forced choice saccadic tasks with scene categories as targets, subjects reached similar performance levels regardless of the target category (natural or man-made environments) with an accuracy rate of respectively 72.2% and 73.8% and a median SRT of respectively 217 and 212 ms (Fig. 2C), values that did not differ significantly between the two types of contexts (accuracy: p = 0.53, median RT: p = 0.47). Furthermore, the minimal SRT was 160 ms for both scene categories (Fig. 2B). Thus, in response to natural and man-made scenes without any foreground objects, saccades were less accurate and initiated later than when participants were targeting animals (accuracy: both p<0.005, median SRT: both p<0.0001). On the other hand, compared with vehicle targets, saccades towards scene categories were more accurate (both p<0.005) with similar median SRT (n.s.) but with minimal SRT values that were shorter with scene category targets.

### Comparison with Computational Models

**Figure 3:**
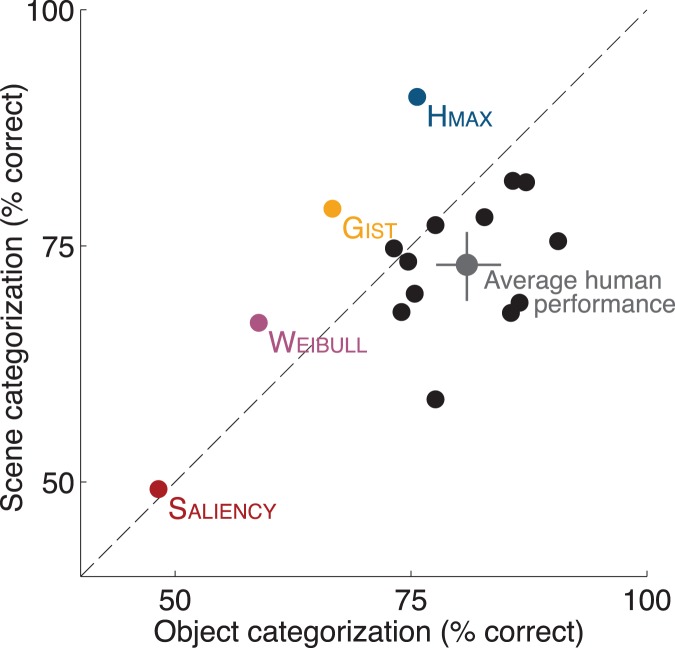
Performance of various computational models and human participants in the object and scene categorization tasks. Colored dots correspond to the performance of the 4 computational models tested, black dots to the performance of individual human participants. The large grey dot illustrates the average human performance (error bars correspond to 95% confidence intervals obtained with bootstrap of the individual participants values).

In order to see if this pattern of results could be derived from standard feedforward computational models, we assessed the performance of four of them: Itti and Koch's Saliency model [22], [23], the Weibull model proposed by the Lamme group [24], [25], Oliva and Torralba’s Gist model [11], [12] and the Hmax model developed in Poggio's group [26]–[28]. They were all shown to be successful in explaining certain characteristics of fast feedforward visual processing [29]. Here, they were tested on the object and scene categorizations with the whole set of images used in the human experiment (see Materials and Methods for details on the testing procedure). As can be seen on Fig. 3, all the models except Saliency performed above chance. This demonstrates that low-level saliency information does not account for the behavioral results observed here and thus validates the image sets. As could be expected from the visual feature complexity of each model, Hmax has clearly the best level of performance, followed by the Gist and the Weibull. But the striking result here is that, in contrast to the performance of human participants, all 3 models were clearly better at classifying scene than object categories. This result shows that human performance cannot be accounted by straightforward visual statistic differences in the image sets, and then support the idea that primate visual processing might be biased toward particular object categories or visual features (such as those corresponding to animals).

### Contextual Influences on Object Processing

**Figure 4:**
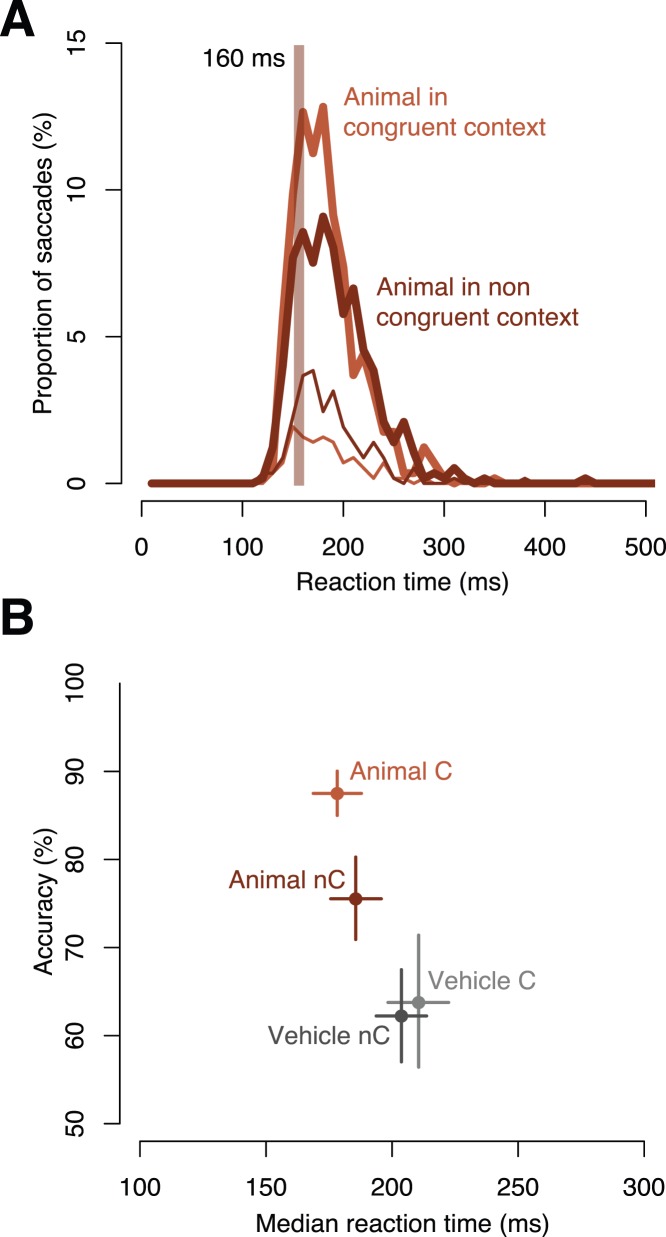
Behavioral results in object tasks when both target and distractor were either congruent (C, light) or incongruent (nC, dark). (A) SRT distributions in the animal task show a higher peak of correct responses (thick lines) and a lower peak of incorrect responses (thin lines) when the context is congruent (vs. incongruent). A c2 test computed on Hits minus FA distributions revealed a difference between congruent and non-congruent responses starting at 160 ms indicated by the grey thick vertical line.(B) Accuracy as a function of median SRT shows a strong congruency effect in the animal task, an effect that is not seen in the vehicle task. Animals in congruent natural contexts are detected more accurately and faster than in incongruent manmade contexts.

As for previous studies [18], [20], the congruence between the object and its context was defined in terms of superordinate categories: animals were considered as congruent in natural contexts while vehicles were congruent in manmade contexts. The aim of this analysis was to know whether object and context processing can interact at very short latencies and to determine the earliest latency for such interactions. Animal and vehicle targets were presented equally in natural or man-made scenes and context influences were computed on trials where both simultaneously presented scenes (target and distractor) were either congruent or incongruent. No difference was observed between congruent and incongruent conditions in the vehicle task since correct saccades had the same accuracy and were triggered with similar median RTs (Fig. 4, respectively, 63.8% vs. 62.2%, n.s. and 210 ms vs. 203 ms, n.s.). On the other hand, correct saccades towards animals were performed more accurately in the congruent vs. incongruent condition, albeit with similar median RTs (respectively, 87.5% vs. 75.5%, p = 0.002 and 178 ms vs. 186 ms, n.s., see individual results Table S2). The higher accuracy when animals are embedded in congruent contexts is also visible in the SRT distribution (Fig. 4A) that had a higher peak of correct responses and a lower rate of incorrect responses in the congruent condition (vs. incongruent). To define the earliest latencies at which this context congruency effect occurs, we computed for each of the two conditions, a single SRT distribution by subtracting false alarm SRTs from hit SRTs, and then compared the two congruent and non-congruent conditions using χ2 tests for each 10 ms bin (SI Methods). While no difference was observed for the vehicle task over the whole range of saccadic response time, animals were better categorized when embedded in a congruent natural context and this effect was observed as early as 160 ms. Interestingly, this value happens to correspond to the minimal SRT obtained in the two context saccadic choice tasks.

## Discussion

The first aim of the present study was to compare the time-courses of object and scene visual processing using a saccadic choice task that allows the investigation of behavioral effects that can occur during an early temporal window, starting from only 120 ms after stimulus onset. The results showed that animal detection clearly precedes the access to scene category. Note that “precede” should not be understood as a necessary first step to access scene category but just as a temporal advantage. This advantage in processing time for animal-objects is at odds with the commonly held view that global scene statistics can be accessed faster than object representations and could even underlie object processing in fast categorization tasks. Our results also suggest that the animal category, in the same vein as the face category, might be a special object category. Finally, the time needed to access scene category is remarkably similar to the time when context starts to modulate the responses toward animal targets (inducing an advantage for congruent natural context) suggesting that the extraction of global scene statistics does not affect object processing prior to this 160 ms latency.

### Asymmetry between Animal and Vehicle Targets

The results of the present study replicated the earlier results obtained by Kirchner & Thorpe [3], by showing that reliable saccades toward animal targets can be initiated in just 120–130 ms. These very short latencies fit with a number of recent imaging studies that have reported that animate object categories are particularly easy to derive on the basis of activity in higher order visual areas in both monkeys [30] and humans [31]. This study also extends the results obtained by Crouzet et al. [4] by demonstrating an asymmetry in targeting different object categories during a saccadic forced-choice task. Using human face and vehicle target categories, Crouzet et al. [4] demonstrated that the very fast saccades towards human faces were not completely under top-down control because they still show a strong bias towards faces even when subjects were instructed to target vehicles. The present study extends this asymmetry to animals and vehicles by revealing a specific and early pattern of errors towards animal distractors when the task requires saccading towards vehicles, an effect not observed with slower manual responses [32]. An analysis of the two sets of object categories (see material and methods) showed that both the animal and the vehicle categories included a wide range of different exemplars, sizes, and eccentricities. This makes the large advantage of the animal category over the vehicle category difficult to explain using a simple search strategy based on a simple set of features. One possibility is that processing of certain key biological stimuli such as animals (and faces) could involve faster hard-wired neural mechanisms possibly tuned by expertise or even by ancestral priorities [4], [7].

The existence of a very rapid mechanism for finding animals raises a number of questions with respect to the extensive literature on visual search [33], [34]. Finding animals in natural scenes appears to be different to the standard situation in visual search where subjects are required to look for a particular combination of features (a green “T”, for example). Given the huge range of possible visual features that can be associated with an animal, it seems implausible to imagine that subjects performing the task can pre-activate all the possible features that could be used to detect the presence of an animal. The existence of a particularly fast mechanism for detecting animals is also suggested by the fact that humans can make fast and accurate saccades to animals in natural scenes, even when no information is provided about the range of possible positions [35]; another evidence that animals constitute a special class of stimulus that does not fit easily with the conventional approach to visual search.

Interestingly, although animals can be detected without attention [36], , they do not pop-out in a visual search display [38], [39]. The preattentive processing could be accounted for by the existence of two feature binding processes [39], [40]. With over-learned categories (such as animal or faces) feature binding would rely on automatic and hard-wired processes up to object selective neuronal populations, as proposed in computational models such as Hmax [27]. On the other hand, when object categories have not been over-learned (such as artificial stimuli often used in standard visual search tasks), feature binding would require attention.

### Scene Category is Extracted Rapidly, but Later than Animal Objects

As mentioned earlier, our results show that human subjects can reliably determine whether the scene is man-made or natural from as early as 160 ms after scene onset. To the best of our knowledge, such short behavioral latencies for scene processing have never been reported before. This relatively straightforward scene distinction is in agreement with previous studies [10], [21] and could be based on global orientation and spatial frequency information [11], [12], [41]. Since finer representations of the scenes (indoor, outdoor, mountains, sea etc) require longer processing times [21], [42], access to more detailed scene concepts at such short latencies seems unlikely.

Although the extraction of scene category is very rapid, an important result of this study is that access to the representation of animals can be even faster. Given that saccades to faces can be triggered at even shorter latencies, it seems likely that processing of faces will, like animals, fall into a special category of objects for which the processing of global scene statistics does not seem to be fast enough to provide any real advantage. The effect reported here between scene and animal-objects is large, with a latency shift of 30–40 ms that cannot be explained by any of the standard feedforward computational models we tested. It clearly demonstrates that the earliest saccades observed towards animal-targets cannot be based on an early access to global scene statistics. This observation is consistent with recent results with objects (animal targets and distractors) pasted on various background [20], for which the Gist model was at chance level. Taken together, the data suggest that rapid animal detection might be performed through a rapid feedforward process based on an hardwired extraction of specific animal features that have been given an advantage, possibly as a result of evolutionary pressure [4], [7]. One possible explanation of this advantage in temporal dynamics could lie in the size of the receptive fields of the neurons involved in analyzing global scene statistics. Whereas it is easy for a computer algorithm to integrate information from across the visual field, we know from neurophysiological data that receptive fields large enough to take into account information from across the image to integrate global scene properties would have to be relatively far forward in the ventral visual pathway – i.e. in the human equivalent of monkey inferotemporal cortex. In contrast, the detection of the critical intermediate features needed to find a specific object such as a face or an animal [6], [29], [43] could potentially be done earlier in the visual system in areas such as V4. Because extrastriate cortical areas including V1, V2 and V4 have direct projections to superficial layers of the superior colliculus [44]–[46], critical animal features could potentially allow very fast saccades to be triggered even before the information has propagated as far as inferotemporal cortex. Thus, for any object that could be detected directly on the basis of neuronal selectivity in areas like V4, it may be possible to trigger saccades before the global scene statistics have been extracted. This could explain why the animal selective saccades can be produced 30–40 ms before the scene category is available. Note, however, that for most other objects (vehicles, furniture, tools, etc), this very fast route may not be available. For these more general cases the scene and object categories may be analyzed at roughly the same level in the visual system, with ample time for interactions.

### Contextual Modulation of Animal Detection

Contextual information starts to bias animal responses (but not vehicle ones) as early as 160 ms after stimulus onset. This latency fits remarkably well with the first latency at which scene category can be extracted. Saccadic accuracy towards animals after this latency was significantly higher when animals were presented in a congruent (vs. incongruent) context. Considering the time constraints of our tasks, this congruency effect is very unlikely to be due to a semantic interference. This view is supported by the fact that, as a post-hoc analysis, when we divided the images of animals in man-made environments into two groups, ones where the animal was in a more “normal” manmade environment (a dog in a street, or a rhino in a zoo, n = 55) and ones where the context was highly unusual (an elephant walking among houses or the head of a giraffe through kitchen window, n = 41), there was no significant differences in either accuracy or mean reaction time (respectively, 75.6% vs. 75.4%, n.s., 185 ms vs. 187 ms, n.s.). This observation needs to be discussed in relation to the functional isolation model proposed by Henderson and Hollingworth [47], [48] which proposes that object and context information are processed independently without interfering. The present results suggest that at the very earliest latencies, and specifically for animal (and probably face) targets, there is indeed a moment when object processing for such specific categories is effectively independent of context. However, contextual effects can be seen within a few tens of milliseconds, and are clearly visible for saccades triggered with SRTs over 160 ms. They could be strengthen if animal categorization had to be performed at a more detailed (basic) levels as such representations are accessed later than superordinate ones [49]. We can speculate that the facilitation/interference effects observed in the present study could reflect the pattern of connectivity set up by experience-dependent synaptic weight modification [50]. Facilitation would reflect repetitive coactivation of selective neuronal responses whereas interference would arise from conflictual co-activations [18], [51]. These co-activation effects, both positive and negative, could potentially take place during the initial wave of feed-forward processing within the ventral visual pathway [18], [51], [52] and could be critical for decision-making, even in the absence of any conscious strategy. Alternatively, object feature integration in the infero-temporal cortex could also be influenced by two kinds of top-down modulations based on global, low-spatial-frequency information: a representation of the contextual frame generated by the parahippocampal cortex and a representation of potential candidate objects originating in the prefrontal cortex [53]. The orbitofrontal cortex has been shown to influence the fusiform gyrus at around 130 ms after stimulus onset [53] a latency that fits with the apparition of the earliest congruency effects in our task if we allow around 30 ms for the effects of such modulations to become visible in the behavioral response.

The absence of a congruency effect for the vehicle responses suggests that the influence of context differs from one category to another [53]. Although animals are mostly associated with natural contexts, vehicles objects (although man-made) are probably less strongly associated with specific man-made context. Indeed boats are equally likely to be seen in a port or on the open sea, and cars and planes are also often seen in purely natural contexts. For this reason, it would be interesting to contrast the animal category with other manmade categories more strongly associated with a particular context such as “furniture” that are usually seen indoors.

Nevertheless, using the saccadic choice task to explore this early window of visual processing clearly demonstrated that access to global scene statistics does not precede access to object superordinate representation and might even in some cases such as animal or face be accessed after a considerable delay. Comparing the performance of models with those of human subjects showed that object detection cannot be based solely on the extraction of global scene statistics. Such data will have to be taken into account in models of object recognition.

## Materials and Methods

### Ethics Statement

This study obtained ethics approval from the CNRS Ethical committee (Institutional review board) and from the “Comité de Protection des Personnes” (South-West Regional Review Board) registered under the number: ID-RCB: 2011-A01621-40. All subjects volunteered and gave their written informed consent to participate in the experiment.

### Participants

12 volunteers (9 females, mean age 21, range 18–26, 10 of them right-handed) gave their written informed consent and had normal or corrected-to-normal vision.

### Stimuli

Altogether, 864 photographs of natural scenes were used in this experiment (see Figure 1) of which 96 were used for training and 768 in the testing blocks. The original images were selected from a large commercial CD-Rom library (Corel Stock Photo Libraries) or from the web. They were cropped to 400×400 pixels, converted to grey-level and their global luminance and RMS contrast were normalized by taking the average luminance and contrast of the whole set. Stimuli were divided into two main sets (Figure 1): scenes that did not contain any foreground object (216 Manmade and 216 Natural), and scenes containing an object (216 Animal and 216 Vehicle). The Animal and Vehicle images were carefully selected so that the surrounding contexts were half manmade and half natural. Images were thus unmodified, in the sense that the objects were never pasted onto the background, and only original images were used. In both categories the exemplars were very varied: for the animal category there were 72% mammals, 14% birds, 8% reptiles, 5% fish including sea mammals and 1% others, whereas for the vehicle category the distribution was 32% cars, 36% ships, 19% planes, 9% trains, 2% bikes and 2% others. The images could contain one or more targets. In order to check that the observed results could not be accounted for by object size or eccentricity in the picture, objects (animals and vehicles) in these images were all delineated manually, their size and centroid position were quantified (Fig. S1). The natural environment images included photographs of coasts, mountains, fields, forests, deserts, icebergs, lakes and savannahs, while the man-made environments included photographs of streets, places and buildings from all around the world using outdoor, indoor views and some aerial views.

### Procedure

Participants sat in a dimly lit room with their head position constrained by a chin rest and a forehead support. Photographs were presented on a computer screen (19″, resolution 1024×768, vertical refresh: 100 Hz) on a mean grey background at a distance of 60 cm resulting in an image size of 14°×14° and a horizontal eccentricity of 8.6°. Image presentation was carried out using Matlab and the Psychophysics Toolbox 3 [54].

On each trial, after a fixation cross displayed for a pseudo-random time interval (800–1600 ms) to avoid anticipations, two natural scenes (one target and one distractor) were presented for 400 ms in the left and right hemifields (see Fig. 2A) centred at 8.6° of eccentricity. Subjects were asked to make a saccade as fast as possible to the side of the target. The trial ended by a 400 ms blank screen before the next trial started.

Eye movements were monitored with an IView Hi-Speed eyetracker (SensoMotoric Instruments, Berlin, Germany), an infrared tracking system that samples eye position at 240 Hz. Saccade detection was performed off-line using the saccade based algorithm of the SMI BeGaze Event Detection package. Before each run, a 13-point calibration was performed. We considered as saccadic reaction times (SRT) the latency of the initiation of the first saccadic response if entering one of the 2 images. SRTs below 80 ms and over 800 ms, if any, were discarded. The number of trials with no data (discarded or no saccade) over the 12 subjects was 1.9±2.4% for the Natural condition, 3.8±6.8% for ManMade, 0.9±1.1% for Animal and 1.8±2.2% for Vehicle. This corresponds to a mean percentage of 2.4% (sd = 2.9, min = 0.08%, max = 10.5%).

The target category was specified at the start of each block, and targets were equiprobable in both hemifields. Overall, the participants performed 4 blocks of the saccadic choice task, and each block included 4 runs of 48 trials preceded by 24 trials of training. During the experiment, each subject performed 2 blocks of context discrimination in which the scenes without foreground objects were used. In one of the blocks the targets were natural scenes whereas in the other block, the targets were man-made scenes. The two remaining blocks used another set of images which contains object in the foreground, and required subjects to target a given object category regardless of its context. One block used animal targets vs. vehicle distractors, while the other used vehicle targets vs. animal distractors. In each of this testing blocks, 50% of the targets and distractors were embedded in a man-made scene, the other half in a natural scene. Each picture was seen twice by each subject, once as target and once as distractor and the pairing of the target and distractor stimuli was random. Task order and presentation hemifield of the stimuli were counterbalanced across subjects.

### Statistics

To estimate the statistical significance of performance differences observed between two conditions both in terms of global accuracy and median SRT, we used a bootstrap method based on Monte Carlo simulations using the following procedure. For each pair-wise comparison, the individual data for the 12 subjects in each of the two conditions was pooled together, randomly shuffled, and redistributed in fake samples with the same sizes as the original samples. The differences between these two new fake populations were then stored. This procedure was run 2000 times, providing a normal distribution based on the null hypothesis that the two conditions were actually sampled from the same population. The p-value was computed by evaluating the number of theoretical differences more extreme than the experimental one.

We also defined a “minimum SRT”. Using a 10 ms time bin SRT distribution, minSRT corresponds to the first bin in which correct responses significantly outnumber errors using a χ^2^ test with a criterion of p<.05 when followed by 4 consecutive bins also reaching this criterion.

To compare speed of performance in congruent and non-congruent conditions, we first computed for each of the two conditions, a single SRT distribution by subtracting false alarm SRTs from hit SRTs. Then, to determine the earliest latency at which a congruency effect (if any) was observed, the two distributions were statistically compared using the same χ^2^ test procedure.

### Computational Models Simulations

To assess the performance of various computational model of visual processing in the object and context tasks, a procedure identical to Crouzet & Serre [29] was used. It runs as follows: first, the image dataset was split in a training set (180 images) and a test set (12 images) that contained an equal proportion of target (90 for training, 6 for testing) and distractor images. Second, an optimal cost parameter *C* was determined through line search optimization using 8-fold cross-validation on the training set of images. A linear SVM classifier [55] was then trained and tested on the given model feature vector (the number of features depended on the model considered, see [29]). For each model, the reported results correspond to the average performance using a cross-validation procedure (n = 100) whereby different training and test sets were selected each time at random.

## Supporting Information

Figure S1 **Characteristics of the animals and vehicles in the natural images.** Every animal and vehicle was manually delineated on all natural images used in the experiment. (A) Geometric centroids of the object(s) obtained for each image of the 2 object categories (animals in red, vehicle in gray). When an image contained multiple objects, the average of their centroid was computed. (B) Average object size (% of the image) for animals (red bar) and vehicles (gray) with corresponding 95% CI.
(EPS)

Table S1 Individual results (accuracy and median RT) of participants in the two tasks (two target classes per task).
(DOC)

Table S2 Individual results (accuracy and median RT) of participants in the object task, while objects were presented in congruent or incongruent context.
(DOC)
