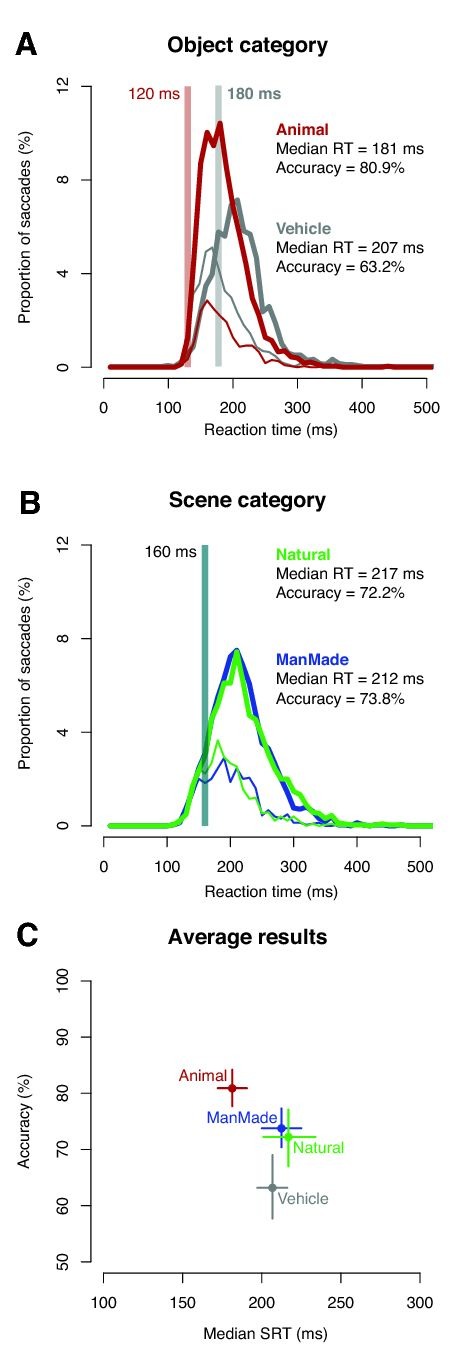# Correction: Animal Detection Precedes Access to Scene Category

**DOI:** 10.1371/annotation/9cc5295b-d8b7-482a-8940-eaa4c8b53a1e

**Published:** 2013-12-16

**Authors:** Sébastien M. Crouzet, Olivier R. Joubert, Simon J. Thorpe, Michèle Fabre-Thorpe

A curve is missing from Figure 2B. Please see the corrected Figure 2 here: 

**Figure:**