# Supplementary material for: Animal Detection Precedes Access to Scene Category
Source: PLoS One. 2012 Dec 10;7(12):e51471. doi: 10.1371/journal.pone.0051471 (PMC3518465; doi:10.1371/journal.pone.0051471)
Supplement: Table S1 — Individual results (accuracy and median RT) of participants in the two tasks (two target classes per task). (DOC) [file pone.0051471.s002.doc]

|  | **GLOBAL IMAGE PROPERTIES** | | | | **OBJECT** | | | |
| --- | --- | --- | --- | --- | --- | --- | --- | --- |
|  | **Manmade** | | **Natural** | | **Animal** | | **Vehicule** | |
| **Participant** | Accuracy (%) | Median RT (ms) | Accuracy (%) | Median RT (ms) | Accuracy (%) | Median RT (ms) | Accuracy (%) | Median RT (ms) |
| 1 | 77 | 226 | 86.5 | 274 | 87.2 | 215 | 82 | 226 |
| 2 | 76.3 | 227 | 74.7 | 231 | 90.6 | 176 | 69.3 | 204 |
| 3 | 67.7 | 188 | 70.3 | 182 | 86.5 | 163 | 51.3 | 187 |
| 4 | 62.8 | 202 | 54.7 | 181 | 77.6 | 172 | 54.7 | 184 |
| 5 | 83.9 | 201 | 79.9 | 198 | 85.8 | 181 | 61.4 | 205 |
| 6 | 71.2 | 216 | 64.6 | 211 | 85.6 | 186 | 52.3 | 215 |
| 7 | 70.5 | 242 | 85.6 | 242 | 82.8 | 188 | 70.5 | 229 |
| 8 | 77.8 | 222 | 76.6 | 243 | 77.6 | 198 | 67.7 | 229 |
| 9 | 76.5 | 256 | 63.4 | 251 | 75.4 | 193 | 52.7 | 216 |
| 10 | 71.7 | 193 | 75 | 191 | 74.7 | 153 | 58.9 | 175 |
| 11 | 67.2 | 179 | 68.8 | 198 | 74 | 165 | 58.6 | 198 |
| 12 | 82.8 | 199 | 66.7 | 201 | 73.2 | 187 | 78.8 | 215 |
| **Average** | 73.8 | 213 | 72.2 | 217 | 80.9 | 181 | 63.2 | 207 |
