# Supplementary material for: Animal Detection Precedes Access to Scene Category
Source: PLoS One. 2012 Dec 10;7(12):e51471. doi: 10.1371/journal.pone.0051471 (PMC3518465; doi:10.1371/journal.pone.0051471)
Supplement: Table S2 — Individual results (accuracy and median RT) of participants in the object task, while objects were presented in congruent or incongruent context. (DOC) [file pone.0051471.s003.doc]

|  | **Animal Object** | | | | **Vehicule Object** | | | |
| --- | --- | --- | --- | --- | --- | --- | --- | --- |
|  | **Congruent Context (Natural)** | | **Incongruent Context (Manmade)** | | **Congruent Context (Manmade)** | | **Incongruent Context (Natural)** | |
| **Participant** | Accuracy (%) | Median RT (ms) | Accuracy (%) | Median RT (ms) | Accuracy (%) | Median RT (ms) | Accuracy (%) | Median RT (ms) |
| 1 | 89.4 | 218 | 87.2 | 210 | 87.2 | 233 | 77.1 | 226 |
| 2 | 97.9 | 178 | 87.5 | 184 | 66.7 | 201 | 62.5 | 203 |
| 3 | 93.8 | 158 | 83.3 | 165 | 53.2 | 199 | 56.2 | 186 |
| 4 | 85.4 | 169 | 66.7 | 175 | 45.8 | 181 | 50 | 181 |
| 5 | 89.4 | 167 | 72.3 | 177 | 59.6 | 206 | 59.6 | 209 |
| 6 | 84.4 | 180 | 85.4 | 202 | 50 | 220 | 61.4 | 217 |
| 7 | 87.5 | 184 | 79.2 | 196 | 74.5 | 243 | 79.2 | 224 |
| 8 | 83.3 | 186 | 64.6 | 216 | 64.6 | 231 | 67.4 | 220 |
| 9 | 80.9 | 195 | 76.6 | 191 | 56.5 | 228 | 52.1 | 204 |
| 10 | 87.5 | 152 | 70.2 | 157 | 54.2 | 172 | 58.3 | 168 |
| 11 | 83.3 | 168 | 64.6 | 166 | 63.8 | 205 | 52.2 | 193 |
| 12 | 87.2 | 185 | 68.8 | 189 | 89.1 | 209 | 70.8 | 215 |
| **Average** | 87.5 | 178 | 75.5 | 186 | 63.8 | 211 | 62.2 | 204 |
